# Supplementary material for: Effect of Reactive Oxygen Scavenger N,N′-Dimethylthiourea (DMTU) on Seed Germination and Radicle Elongation of Maize
Source: Int J Mol Sci. 2023 Oct 25;24(21):15557. doi: 10.3390/ijms242115557 (PMC10649595; doi:10.3390/ijms242115557)
Supplement: Supplementary file 1 [file ijms-24-15557-s001.zip › Supplementary materials Table S2.pdf]

Effect of Reactive Oxygen Scavenger N,N'-Dimethylthiourea (DMTU) on Seed Germination and Radicle Elongation of Maize

Wei-Qing Li [1], Jia-Yu Li [1], Yi-Fei Zhang [1,2,*], Wen-Qi Luo [1], Yi Dou [1] and Song Yu [1,2]

Table S2 Gene-specific primers for qRT-PCR

Gene Name	Forward (5′ to 3′)	Reverse (5′ to 3′)
ZmSOD4	GATCTTGGAAAGGGTGGACA	GAAGTCCAGCGACCCATTT
ZmAPX2	CCCATCCTATCCTACGCTGA	ATCAGGTCCGCCGGTTAC
ZmCAT2	ACGACATCACCCACCTGAC	GGAGAAGCGGACGATGAC
ZmActin	TACGAGATGCCTGATGGTCAGGTCA	TACGAGATGCCTGATGGTCAGGTCA
